# PB.26. MRI-guided breast biopsy in Leeds: 12 years' experience

**DOI:** 10.1186/bcr3709

**Published:** 2014-11-03

**Authors:** S Rajan, N Sharma, B Dall

**Affiliations:** 1 Breast Imaging Unit, St James's University Hospital Trust, Leeds, UK

## Introduction

MRI-guided biopsy allows sampling of lesions occult on conventional imaging to reach a preoperative histological diagnosis and guide patient management.

## Methods

We performed a retrospective audit of all MRI-guided biopsies performed in Leeds patients over a 12-year study period from the initiation of this service in April 2002. Data collected: indication for MRI; pre-biopsy MRI score; histology from MRI-guided biopsy and final surgery.

## Results

There were 46 MR-guided breast biopsies performed (symptomatic *n *= 26, screening *n *= 20). Indication for MRI was to assess extent of malignancy (*n *= 29), an adjunct to triple assessment (*n *= 11) and a screening investigation in higher risk patients (*n *= 6). Pre-biopsy MRI score was classified as indeterminate MRI 3 (*n *= 22), suspicious MRI 4 (*n *= 20) and malignant MRI 5 (*n *= 4). In the MRI 3 subgroup, five proved malignant at MR-guided biopsy with a positive predictive value of 23% (5/22). In the MRI 4/5 subgroup, 14 proved malignant at MR-guided biopsy and a further four malignant lesions were identified at diagnostic surgical biopsy, with an overall positive predictive value of 75% (18/24).

## Conclusion

MRI-detected lesions are interpreted in clinical context and attributed an MRI score. If the patient has an increased risk of cancer or there already is a cancer present, any additional lesion should be regarded with a higher level of suspicion. MRI 4/5 lesions have a high positive predictive value for malignancy and should proceed to diagnostic surgical biopsy if the initial MR-guided biopsy is inconclusive.

